# Factors Affecting the Foveal Avascular Zone Area in Healthy Eyes among Young Chinese Adults

**DOI:** 10.1155/2020/7361492

**Published:** 2020-03-24

**Authors:** Yifan Zhou, Minwen Zhou, Min Gao, Haiyun Liu, Xiaodong Sun

**Affiliations:** ^1^Department of Ophthalmology, Shanghai First People's Hospital, School of Medicine, Shanghai Jiao Tong University, Shanghai, China; ^2^Shanghai Key Laboratory of Fundus Disease, Shanghai, China

## Abstract

**Purpose:**

To evaluate the influence of systemic and ocular factors on the foveal avascular zone (FAZ) area in young Chinese subjects' healthy eyes.

**Methods:**

The current observational, cross-sectional study included 344 eyes from 172 healthy individuals (103 women, 69 men). Optical coherence tomography angiography realized with the split-spectrum amplitude-decorrelation angiography (SSADA) algorithm was used to assess the area of superficial FAZ. To determine the related factors and to reveal their potential correlations with the FAZ area, comprehensive examinations including both systemic and ocular ones were executed. Systemic examination involved factors including age, gender, and body mass index, while ocular examination involved factors including BCVA, refractive error, intraocular pressure, axial length (AL), anterior chamber depth, and central corneal thickness. Especially for fundus examination, central macular thickness (CMT), retinal volume, mean retinal thickness, macular blood flow area/vessel density in the superficial retinal layer (SRL) and deep retinal layer (DRL), mean retinal nerve fiber layer (RNFL) thickness, ganglion cell layer (GCL) thickness, C/D rate, rim area, and subfoveal choroid thickness were assessed, using mixed-effects regression models to appropriately account for intereye correlation. Subgroup analyses were performed based on gender and high myopia categories.

**Results:**

The mean FAZ area was 0.30 ± 0.11 mm^2^ and varied significantly across gender (*P* = 0.0024). AL, CMT, and RNFL thickness were found significantly correlated with the FAZ area in the univariate regression analysis (AL, *P* = 0.0005; CMT, *P* < 0.0001; and RNFL thickness, *P* = 0.0461). According to the multivariate results, CMT and macular blood flow in SRL were negatively correlated with FAZ (CMT: *P* < 0.0001; macular blood flow in SRL: *P* = 0.00223). Mean retinal thickness, mean GCL thickness, and macular blood flow in DRL were positively correlated with FAZ (mean retinal thickness: *P* = 0.0005; mean GCL thickness: *P* < 0.0001; and macular blood flow in DRL: *P* = 0.0099). Correlation results among these filtered factors and FAZ were more pronounced in non-high-myopic eyes than in high-myopic eyes and had a significant difference when data of male and female subjects were processed separately from each other.

**Conclusion:**

The present cross-sectional study performed comprehensive systemic and ocular examinations in young Chinese adults and filtered factors affecting FAZ. We indicated that among all the assessed candidate factors, gender, AL, retinal thickness, macular blood flow, RNFL, and GCL thickness affected the FAZ area most significantly. Such findings would facilitate future research concerning the role of FAZ variation in fundus diseases.

## 1. Introduction

Retinal vasculature is essential for evaluating retinal conditions and also for predicting the progression of some vision-threatening retinal diseases such as diabetic retinopathy (DR), glaucoma, age-related macular degeneration (AMD), and retinal vein occlusions (RVO) [1–3]. Accurate *in vivo* measurement of retinal blood flow can be used to monitor some retinal diseases. Among these parameters, the foveal avascular zone (FAZ) is a specialized capillary-free area in the central macula and it is in proximity to the region of the highest cone photoreceptor density and oxygen consumption [4], which enjoys unparalleled significance. As such, variations in the area of the FAZ may be associated with visual function. More importantly, these changes can be of both diagnostic and prognostic value in various retinal disorders [5–8].

To determine the relationship between variation in the FAZ area and retinal disorder, it is essential to first realize the variation in the FAZ area and factors affecting FAZ in healthy individuals. To date, numerous FAZ-related studies have been reported; however, to realize factors affecting the FAZ area and potential correlations, most studies artificially selected only a small number of programs like gender, axial length, retinal construction, and vasculature parameters [9–14]. To our knowledge, there is very little research in which comprehensive examinations including both systemic and ocular ones were executed to determine factors affecting the FAZ area. We considered that lack of a highly profound profile of examinations would compromise statistical results, so comprehensive examinations were necessary to perform more practical results. To our knowledge, there has not been any relative FAZ study with a large sample aiming at filtering factors affecting FAZ and FAZ variations in our young adults. Therefore, there is a need for our study to provide detailed analysis in the present article.

## 2. Methods

### 2.1. Subject Enrollment

This observational, cross-sectional study was performed with the approval of the Ethical Review Committee of Affiliated First People's Hospital of Shanghai Jiao Tong University. All subjects were treated according to the principles of the Declaration of Helsinki. Written informed consent was obtained, and an evaluation of demographic information, medical history, and anthropometric examinations was carried out. Subjects with any of the following criteria were excluded: (1) unable to tolerate an OCT angiography (OCTA) examination, (2) failure to maintain stable fixation leading to low-quality images (signal strength index (SSI) < 50), (3) best-corrected visual acuity (BCVA) < 20/20, and (4) history of ocular disease (except for ametropia) or history of ocular surgery or any systemic diseases such as hypertension and diabetes mellitus.

### 2.2. Systemic and Ocular Examinations

**Figure 1:**
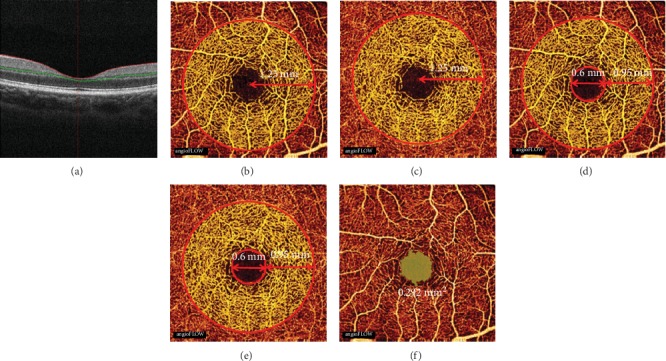
*En face* optical coherence tomography (OCT) angiograms (3 × 3 mm) of the macula in representative young Chinese subjects. *En face* representation of the parafoveal blood flow area in the superficial retinal layer (SRL) (a) and the deep retinal layer (DRL) (b) in a circular zone with a radius of 1.25 mm centered on the foveal avascular zone (FAZ). *En face* representation of the parafoveal vessel density in the SRL (c) and the DRL (d) in an annular zone with an outer diameter of 2.5 mm and an inner diameter of 0.6 mm centered on the FAZ. (e) This young Chinese subject's eye had a FAZ of 0.292 mm^2^. (f) Typical cross-sectional image.

Basic clinical characteristics include age, gender, and body mass index (BMI). All subjects underwent a thorough ophthalmic examination that included BCVA, refractive error, intraocular pressure (IOP) measurement, axial length (AL), anterior chamber depth (ACD), and central corneal thickness (CCT). Fundus exams used the Fourier-domain OCT system (RTVue-XR, Optovue, Fremont, CA, USA) to obtain images. Both macular and retinal nerve areas were scanned, which included central macular thickness (CMT), retinal volume, mean retinal thickness, macular blood flow area/vessel density in the superficial retinal layer (SRL) and deep retinal layer (DRL), mean retinal nerve fiber layer (RNFL) thickness, ganglion cell layer (GCL) thickness, C/D rate, rim area, and subfoveal choroid thickness (SFCT). Most of the data listed above was automatically generated by machines. Macular blood flow area/vessel density and FAZ area were determined from the *en face*3 × 3 mm image of the macula. The macular blood flow area was defined as a circular region with a radius of 1.25 mm centered on the FAZ (Figures 1(b) and 1(c)) and was calculated as the area occupied by the large vessels and microvasculature in the defined region. Macular vessel density was defined as an annular zone with an outer diameter of 2.5 mm and an inner diameter of 0.6 mm that was centered on the FAZ and calculated as the percentage area occupied by the large vessels and microvasculature in the defined region (Figures 1(d) and 1(e)). The FAZ area was outlined and measured in images magnified six times (Figure 1(f)). An *en face* angiogram of the retinal circulation was segmented from the inner limiting layer to the retinal epithelial pigment, by which we could measure the macular blood flow area/vessel density in the SRL and DRL. SFCT is measured vertically, at the fovea, from the outer surface of the retinal pigment epithelium (RPE) to the choroid-sclera interface. All these parameters were measured using the software (version 2014.2.0.93) of the OCT machine.

### 2.3. Statistical Analyses

Continuous variables were expressed as mean ± standard deviation or median. If tests of homogeneity of variance and normality were satisfied, the two-sample independent *t*-test was used to compare characteristics between groups. Otherwise, the nonparametric Wilcoxon test was used. For categorical variables, proportions were calculated and the *χ*^2^ test was used for analysis. We used linear mixed model regression analyses for continuous data (FAZ size as the dependent variable) to account for within-person correlation arising from both eyes. Factors associated with FAZ size were identified through both univariate and multivariate analyses. In the final multivariate model, an automatic backward selection strategy was done with the R package “lmerTest” to eliminate all nonsignificant variables, retaining only variables with *P* < 0.05. As gender and high myopia were generally considered to influence the foveal microvasculature [15, 16], we further conducted subgroup analyses based on gender and myopic status in our subjects. High myopia was defined as spherical equivalent ≤ −6.00 Diopter (D).

For all statistical analyses, a *P* value of <0.05 was considered statistically significant. Analyses were performed using the SAS software version 9.4 (SAS Institute, Cary, NC) and R software version 3.4.3.

## 3. Results

### 3.1. Demographic Data and Clinical Characteristics

In total, 176 subjects were originally enrolled for the present study. Of these, one subject was diagnosed with glaucoma and one participant was found to be affected by CNV. Both were excluded from participating in this study. Another two subjects were also excluded due to failure of maintaining fixation stability. Finally, 172 subjects (344 eyes) were included in the study. The mean FAZ area of all eyes was 0.30 ± 0.11 mm^2^. Male subjects (69, 40.12%) had a FAZ area that was, on average, 0.05 mm^2^ smaller than that of the female subjects. The mean age was 25.94 ± 1.92 years for male subjects and 25.98 ± 1.70 for females. All demographic data are shown in Table 1.

### 3.2. Univariate and Multivariate Regression Analyses

We performed comprehensive examinations including both systemic and ocular ones as mentioned above. According to the univariate regression results, we found that gender, AL, CMT, and mean RNFL thickness were significantly related to the FAZ area in our sample. The results of the univariate regression analyses performed for the FAZ area and each variable are displayed in Table 2.

Multivariate regression analysis demonstrated that CMT, mean GCL thickness, mean retinal thickness, and blood flow in SRL and DRL were significantly correlated with FAZ (Table 3). CMT and macular blood flow in SRL were both found to be negatively correlated with FAZ (CMT: *P* < 0.0001; macular blood flow in SRL: *P* = 0.00223). We also noticed that mean retinal thickness, mean GCL thickness, and macular blood flow in DRL were positively correlated with FAZ (mean retinal thickness: *P* = 0.0005; mean GCL thickness: *P* < 0.0001; and macular blood flow in DRL: *P* = 0.0099).

### 3.3. Subgroup Analysis

#### 3.3.1. Gender

As presented in Table 1, male subjects had an FAZ area that was, on average, 0.05 mm^2^ smaller than that of the female subjects. Males also had larger BMI, larger AL but less refractive error, deeper anterior chamber, larger C/D rate with a smaller rim area and thinner RNFL thickness, thicker central macular and subfoveal choroidal thickness, and less blood supply in DRL. Based on the five factors determined by the multivariate regression analysis, we further separated the sample by gender and executed an advanced analysis. We noticed that mean GCL thickness was positively and significantly correlated with FAZ in males, while female subjects displayed a slightly negative correlation between mean GCL thickness and FAZ. Females also had a more pronounced and positive correlation between mean retinal thickness and FAZ (Table 4).

#### 3.3.2. High Myopia

According to our univariate and multivariate results, high myopia was not significantly correlated with FAZ. Indeed, we found that the mean FAZ area in eyes without high myopia was 0.31 ± 0.11 mm^2^, and it was 0.30 ± 0.08 mm^2^ in high-myopic eyes (*P* = 0.745). When it comes to the filtered factors according to the multivariate analysis, in eyes without high myopia, we found that mean GCL thickness was positively and significantly correlated with FAZ, while in high-myopic eyes, it had no significant correlation with FAZ. In normal eyes, blood flow in SRL and DRL also had a significant correlation with FAZ, but it was not the case in high-myopic eyes (Table 4).

## 4. Discussion

Given the indispensable role of the FAZ area as a crucial parameter, understanding the factors affecting FAZ in normal subjects would help us to understand macular disorders. The present study included comprehensive examinations, which to our knowledge is the first to examine the relationships between the FAZ area and factors such as age, gender, BMI, refractive error, IOP, AL, ACD, CCT, macular and optic nerve area structure, and vasculature in one relatively large sample. With excellent completion, the results on factors affecting the FAZ should be realistic and instructive.

Using the OCTA technique, the present study provides *in vivo* evidence that the mean area of the FAZ in the superficial retinal layer among young Chinese adults is 0.30 ± 0.11 mm^2^, which is significantly associated with several clinical characteristics. Various studies have reported different FAZ sizes based on their subjects [11, 14, 17–19]. Reasons for the discrepancy might be attributed to ethnic background, sample size, and different age stages of the previous studies. Thus, our study adds to the current knowledge of quantitative data on the normal FAZ area in the superficial retinal layer among Chinese young adults. According to several of the studies mentioned above, the FAZ area was found to decrease significantly by aging [9, 14]. Combining our data with that from another two studies with large samples in our population, we did find a decreasing trend of FAZ by aging [17, 20]. But we failed to get a significant correlation between FAZ and age in our univariate or multivariate analysis, due to the limited range of age among our subjects. Thus, further studies with enlarged sample size across different age stages are needed to verify age-related variation for FAZ.

Male subjects had an FAZ area that was, on average, 0.05 mm^2^ smaller than that of the female subjects. We did find a significant correlation between gender and FAZ in the univariate regression (*P* = 0.0024). We know that estrogen, androgen, and progesterone receptors are present throughout the eye and that these steroids are locally produced in ocular tissues. Sex hormones can have a neuroprotective role on the retina and could thus modulate ocular blood flow. Women, especially postmenopausal ones, are more likely to suffer from a macular hole, probably owing to the sudden drop in estrogen production in later middle age [21]. However, after adjusting other parameters like age and retinal thickness in the multivariate model, we failed to demonstrate a significant correlation in our young adults.

Some of the literatures indicate that a relatively larger eyeball with larger AL might be a factor contributing to structural and physiological differences [21]. Regardless of other factors, we demonstrated that an increased AL was associated with a smaller FAZ area (*P* = 0.0005). However, when evaluated in multivariate regression, we failed to find a significant correlation. Different studies have concluded controversial results when it comes to relation between AL and FAZ [10, 12, 14, 15, 17, 20]. One possible reason of the discrepancy might be explained by the different experimental designs leading to differential statistic results [17, 20]. Tan et al. demonstrated that AL did not significantly affect the FAZ area after adjusting for CMT and gender [10]. Fujiwara et al. found no significant correlation between AL and FAZ as well after adjusting age, gender, CRT, retinal vascular density, and refractive error [14]. Similarly, after adjusting other ocular and systemic factors in our multivariate model, we failed to find a significant correlation between AL and FAZ in our sample.

There were 78 high-myopic eyes (22.67%) from our subjects, which is similar to the prevalence of high myopia among our young population [22]. The elongation of AL may impact vasculature measurements by OCTA, including FAZ [23]. Due to the current tech limit, the inevitable magnification error should be taken into consideration. According to common knowledge, high-myopic eyes usually have poorer fundus construction and less blood supply; thus, they are more sensitive to maculopathy and retinal disorder by aging. Some considered that high myopia could cause severe fundus change in a relatively older age, but the effects are not that pronounced among young people [17]. One possible explanation might lie in the autogenous regulation to ocular ischemia and maculopathy caused by axial elongation. However, in addition to normal examinations in daily medical practices like fundus photograph, RNFL thickness, and visual field test, more refined examinations like multifocal electroretinography (multifocal ERG) and microperimetry in recent studies have indicated that high-myopic-related changes (functional and/or structural) may occur in patients with younger age as compared to what we used to expect [24, 25]. According to the present study, high myopia was not significantly correlated with FAZ, in neither univariate nor multivariate results. However, our subgroup analysis demonstrated that the correlation yielded from multivariate regression analysis on whole eyes was more feasible in non-high-myopic eyes (Table 4). This suggests that preclinical changes which are not easily detectable with the current routine follow-up clinical practices may already exist in young high-myopic eyes. More advanced examinations should be considered for application to follow up visits in high-myopic patients.

Macular blood flow in both SRL and DRL showed a significant correlation with FAZ according to the multivariate results. As for the SRL blood flow, a negative correlation with the FAZ area was revealed, which may be explained by the fact that the parafoveal blood flow area also includes the FAZ area. We reported vessel density in units of mm^−1^, which more accurately reflects perfusion of both retinal capillary plexuses [26]. The superficial and deep vascular plexuses are really different from each other. Indeed, the first one contains big vessels and capillaries; the second one has only capillaries. The capillaries in the DRL are terminated farther from the fovea than those in the SRL, leading to an apparently larger FAZ in the deep layer [10, 27]. As previously reported, the FAZ borders were more clearly delineated at the level of the SRL than at the level of the DRL [11, 28]. So we considered that the negative correlation between blood flow in SRL and the FAZ might be more reliable according to our results.

We found the larger FAZ is associated with thinner CMT in both the univariate and multivariate regression analyses, thus indicating the possible relation between CMT and FAZ, which was demonstrated by numerous studies [10, 12, 14–17, 20]. We may also suggest that when comparing the FAZ area between individuals in clinical research and daily clinic, adjusting for CRT is more important than adjusting for retinal vascular density and other ocular parameters [14]. Together with the significantly negative correlation between blood flow in SRL and FAZ, the results suggested a positive correlation between these two parameters; that is, a thicker retina has higher metabolic requirement (more blood supply), which is associated with the reduction of the FAZ.

CMT remains stable throughout life, but the ganglion cell layer and the retinal nerve fiber layer get thinner by aging [29]. We know that glaucomatous optic neuropathy is characterized by progressive retinal ganglion cell death and axonal loss, especially in open-angle glaucoma (OAG) [30]. Also, blood flow insufficiency and/or impaired microcirculation is thought to play an important role in OAG [31, 32]. So far, relative studies have found consistent results; that is, patients with OAG have a relatively larger FAZ area, along with other macular vasculature changes [33–36]. Some researchers also suggested that variations in FAZ and fundus vasculature have become important indicators for diagnosis and prognosis of OAG [33, 35, 36]. However, to our knowledge, very few studies have revealed the correlation between optic nerve area-related parameters (RNFL and GCL thickness) and FAZ in normal people, or in other words, the potential relations between these glaucomatous parameters and the FAZ area have not been demonstrated. Interestingly, in the present study, we found larger FAZ to be correlated with thicker RNFL thickness based on the univariate regression result. According to the multivariate regression results, mean GCL thickness was positively correlated with FAZ, especially in our male subjects. These findings might suggest the potential role of the enlarged FAZ with GCL and RNFL thinning as an indicator for the diagnosis and prognosis of OAG. Furthermore, other than FAZ variation, the correlation between glaucomatous parameters (RNFL and GCL thickness) and other macular vasculature (like blood flow or vessel density) deserves further studying.

The present study had several strengths. First, there was a relatively large sample size (344 eyes), and the subjects had a wide range of clinical characteristics such as BMI (16.52-30.86) and AL (21.31 mm–29.48 mm), which could increase the accuracy of the regression analyses. Second, in the present study, we indeed performed highly comprehensive systemic and ocular examinations among our subjects. We used linear mixed model regression analyses to account for a within-person correlation arising from both eyes to clarify the possible factors affecting FAZ, ignoring the correlation in previous publications which might lead to invalid inferences [14]. Third, the present study revealed a potential correlation between optical parameters (GCL/RNFL thickness) and FAZ, which has not yet been studied in healthy subjects of large sample size. Fourth, based on gender and myopic status, subgroup analysis revealed significant differences among filtered factors affecting FAZ.

Despite the strengths of this study, its limitations should also be noticed. First, the subjects were relatively young Chinese adults; therefore, generalisability of our study results might be limited by aging and racial reasons. However, we feel that a narrow age range may confer an advantage, since elder subjects may have unperceived systemic worries which might affect the ocular structures and function such as mild vascular sclerosis, early diabetes, and medication history [37], which makes it more complicated to determinate factors affecting FAZ. This could also explain why numerous studies yielded controversial results in terms of age-related FAZ variation [9, 10, 12, 17, 20]. To obtain more generalisable results, further studies with enlarged sample size across different age states are needed. Second, to get more precise calculation of the FAZ area, the scan area is relatively small (3 × 3 mm). We obtained data for the parafoveal but not the overall retina. Larger-area angiograms should be completed in a further study. Third, the causal relationship between the FAZ area and its associated factors cannot be inferred because of the cross-sectional nature of our data.

In conclusion, in this present cross-sectional study, we performed comprehensive systemic and ocular examinations in young Chinese adults and filtered factors affecting FAZ. We found gender, axial length, central macular thickness, and RNFL thickness to be correlated with FAZ according to univariate regression results and GCL thickness, mean retinal thickness, central macular thickness, and macular blood flow to be correlated with FAZ according to multivariate regression results. The correlation results were more pronounced in non-high-myopic eyes than in high-myopic eyes and had a significant difference when applied across gender.

## Figures and Tables

**Table 1:** Demographic and clinical characteristic variables by gender.

	Total	Male	Female	*P* value
FAZ (mm^2^)	0.30 ± 0.11	0.27 ± 0.10	0.32 ± 0.10	0.0001
Age (years)	25.95 ± 1.79	25.94 ± 1.92	25.98 ± 1.70	0.4745
BMI (kg/m^2^)	21.19 ± 2.57	22.84 ± 2.62	20.09 ± 1.92	<0.0001
IOP (mmHg)	16.74 ± 3.13	16.57 ± 3.15	16.92 ± 3.11	0.1237
Refractive error (D)	−3.90 ± 2.61	−3.26 ± 2.44	−4.26 ± 2.67	0.0015
AL (mm)	24.98 ± 1.22	25.15 ± 1.16	24.85 ± 1.26	0.0042
ACD (cm)	3.62 ± 0.27	3.71 ± 0.27	3.57 ± 0.26	<0.0001
CCT (*μ*m)	540.70 ± 34.22	543.73 ± 35.85	538.25 ± 33.41	0.0719
CMT (*μ*m)	244.60 ± 19.61	248.63 ± 19.18	242.50 ± 18.72	0.0020
Retinal volume (mm^3^)	10.16 ± 0.73	10.24 ± 1.02	10.11 ± 0.44	0.3131
Mean retinal thickness (*μ*m)	281.50 ± 12.35	282.33 ± 12.38	280.94 ± 12.13	0.3025
Mean GCL thickness (*μ*m)	83.06 ± 6.50	83.78 ± 4.99	82.60 ± 7.38	0.0574
Mean C/D rate	0.39 ± 0.18	0.43 ± 0.19	0.37 ± 0.17	0.0014
Mean RNFL thickness (*μ*m)	100.80 ± 10.05	98.27 ± 11.26	102.33 ± 8.88	0.0003
SFCT (*μ*m)	272.80 ± 73.17	285.31 ± 72.89	265.24 ± 71.84	0.0091
Blood flow in SRL (mm^2^)	2.96 ± 0.26	2.95 ± 0.25	2.97 ± 0.27	0.3175
Blood flow in DRL (mm^2^)	2.80 ± 0.40	2.72 ± 0.43	2.85 ± 0.37	0.0076
Vessel density in SRL (mm^−1^)	0.63 ± 0.06	0.62 ± 0.06	0.63 ± 0.07	0.3046
Vessel density in DRL (mm^−1^)	0.59 ± 0.10	0.57 ± 0.10	0.60 ± 0.10	0.0036

**Table 2:** Univariate analysis among factors and superficial foveal avascular zone (FAZ) area.

	Estimate	Standard error	*t* value	*P* value
Age (mm^2^)	0.003537	0.00455	0.78	0.4381
Gender	-0.04984	0.01616	-3.08	0.0024
IOP (mmHg)	-0.00027	0.0012	-0.23	0.8195
BMI (kg/m^2^)	-0.00473	0.003116	-1.52	0.1306
Refractive error (D)	0.002868	0.002065	1.39	0.1667
High myopia	-0.005858	0.008022	-0.73	0.4663
AL (mm)	-0.0158	0.004414	-3.58	0.0005
ACD (cm)	-0.01281	0.01632	-0.78	0.4337
CCT (*μ*m)	0.000191	0.000186	1.02	0.3073
CMT (*μ*m)	-0.0024	0.00024	-9.99	<0.0001
Retinal volume (mm^3^)	0.000294	0.003448	0.09	0.9322
Mean retinal thickness (*μ*m)	-0.00011	0.000382	-0.29	0.7723
Mean GCL thickness (*μ*m)	-0.00048	0.000404	-1.19	0.2362
Rim area (mm^2^)	0.006454	0.01362	0.47	0.6364
Mean C/D rate	0.0236	0.02336	1.01	0.3137
Mean RNFL thickness (*μ*m)	0.0006	0.000298	2.01	0.0461
SFCT (*μ*m)	-7.83*E*-06	0.000053	-0.15	0.8824
Blood flow in SRL (mm^2^)	-0.00313	0.009708	-0.32	0.7475
Blood flow in DRL (mm^2^)	0.006743	0.00605	1.11	0.2668
Vessel density in SRL (mm^−1^)	-0.00169	0.03842	-0.04	0.965
Vessel density in DRL (mm^−1^)	0.03006	0.02494	1.21	0.2299

**Table 3:** Multivariate analysis among factors and superficial foveal avascular zone (FAZ) area.

	Estimate	Standard error	*t* value	*P* value
CMT (*μ*m)	-0.0029	0.000275	-10.55	<0.0001
Mean retinal thickness (*μ*m)	0.001424	0.000399	3.57	0.0005
Mean GCL thickness (*μ*m)	0.00138	0.000316	4.37	<0.0001
Blood flow in SRL (mm^2^)	-0.05579	0.02416	-2.31	0.0223
Blood flow in DRL (mm^2^)	0.03974	0.01522	2.61	0.0099

**Table 4:** Subgroup analysis of identified factors according to multivariate regression results.

	Estimate	Standard error	*t* value	*P* value
Female				
CMT (*μ*m)	-0.00392	0.000372	-10.55	<0.0001
Mean retinal thickness (*μ*m)	0.002378	0.000487	4.88	<0.0001
Mean GCL thickness (*μ*m)	-0.00031	0.00046	-0.67	0.5056
Blood flow in SRL (mm^2^)	-0.03861	0.03006	-1.28	0.2022
Blood flow in DRL (mm^2^)	0.02665	0.0205	1.3	0.197
Male				
CMT (*μ*m)	-0.00238	0.000433	-5.51	<0.0001
Mean retinal thickness (*μ*m)	0.00105	0.000662	1.59	0.1188
Mean GCL thickness (*μ*m)	0.002103	0.000464	4.53	<0.0001
Blood flow in SRL (mm^2^)	-0.03236	0.03908	-0.83	0.4113
Blood flow in DRL (mm^2^)	0.02917	0.02212	1.32	0.1928
HM eyes				
CMT (*μ*m)	-0.00429	0.000599	-7.16	<0.0001
Mean retinal thickness (*μ*m)	0.002895	0.000819	3.53	0.0019
Mean GCL thickness (*μ*m)	-0.00094	0.000659	-1.43	0.1672
Blood flow in SRL (mm^2^)	0.03891	0.05534	0.7	0.4894
Blood flow in DRL (mm^2^)	-0.03279	0.0382	-0.86	0.3999
Non-HM eyes				
CMT (*μ*m)	-0.00305	0.000315	-9.68	<0.0001
Mean retinal thickness (*μ*m)	0.00123	0.000462	2.66	0.0089
Mean GCL thickness (*μ*m)	0.001758	0.000367	4.8	<0.0001
Blood flow in SRL (mm^2^)	-0.05841	0.02875	-2.03	0.0446
Blood flow in DRL (mm^2^)	0.04149	0.01746	2.38	0.0193

## Data Availability

No relative data.
